# Touch and go: nuclear proteolysis in the regulation of metabolic genes and cancer

**DOI:** 10.1002/1873-3468.12087

**Published:** 2016-02-18

**Authors:** Laure Maneix, André Catic

**Affiliations:** ^1^ Huffington Center on AgingBaylor College of MedicineHoustonTXUSA; ^2^ Dan L. Duncan Comprehensive Cancer CenterBaylor College of MedicineHoustonTXUSA; ^3^ Stem Cells and Regenerative Medicine CenterBaylor College of MedicineHoustonTXUSA; ^4^ Department of Molecular and Cellular BiologyBaylor College of MedicineHoustonTXUSA

**Keywords:** genome regulation, mitochondria, multiple myeloma, nuclear proteostasis, nuclear receptor corepressor 1, ubiquitin–proteasome system

## Abstract

The recruitment of transcription factors to promoters and enhancers is a critical step in gene regulation. Many of these proteins are quickly removed from DNA after they completed their function. Metabolic genes in particular are dynamically regulated and continuously adjusted to cellular requirements. Transcription factors controlling metabolism are therefore under constant surveillance by the ubiquitin–proteasome system, which can degrade DNA‐bound proteins in a site‐specific manner. Several of these metabolic transcription factors are critical to cancer cells, as they promote uncontrolled growth and proliferation. This review highlights recent findings in the emerging field of nuclear proteolysis and outlines novel paradigms for cancer treatment, with an emphasis on multiple myeloma.

## Abbreviations

**AR** androgen receptor

**ChIP** chromatin immunoprecipitation

**CREB** cAMP response element‐binding protein

**EBPδ** enhancer‐binding protein delta

**GR** glucocorticoid receptor

**HIF‐1α** hypoxia‐inducible factor‐1 α

**IKZF1** Ikaros family zinc finger protein 1

**IKZF3** Ikaros family zinc finger protein 3

**MM** multiple myeloma

**NCoR1** nuclear receptor corepressor 1

**NRF‐2** nuclear respiratory factor‐2

**pVHL** von Hippel–Lindau tumor suppressor protein

**ROS** vreactive oxygen species

**T‐ALL** T‐cell acute lymphoblastic leukemia

**TGF‐β** transforming growth factor‐β

**UPS** ubiquitin–proteasome system

## Integrating metabolism and tumor biology

Over the past decade, extensive work has confirmed the existence of a link between cancer cell‐intrinsic metabolism and tumor signaling pathways. It is now clear that dysregulation of oncogenic signal transduction pathways alters metabolic flux; conversely, mutations in enzymes regulating metabolic flux can initiate cellular transformation and contribute to tumor maintenance and progression 1. Understanding the interconnectivity between dysregulated cell metabolism and tumor biology gives new hope that tumor‐specific metabolic alterations may be used to diagnose and treat malignancies 2.

Recent evidence has emerged that a direct cross‐talk between metabolism and epigenetics is a key regulatory mechanism in cancer development. Specifically, proteome modifications provide a fast mechanism to respond to environmental changes and to adjust homeostasis. Regulated protein catabolism via the ubiquitin–proteasome system (UPS) controls a wide variety of cellular functions, from transcriptional regulation and stress response to cell cycle regulation.

**Figure 1:**
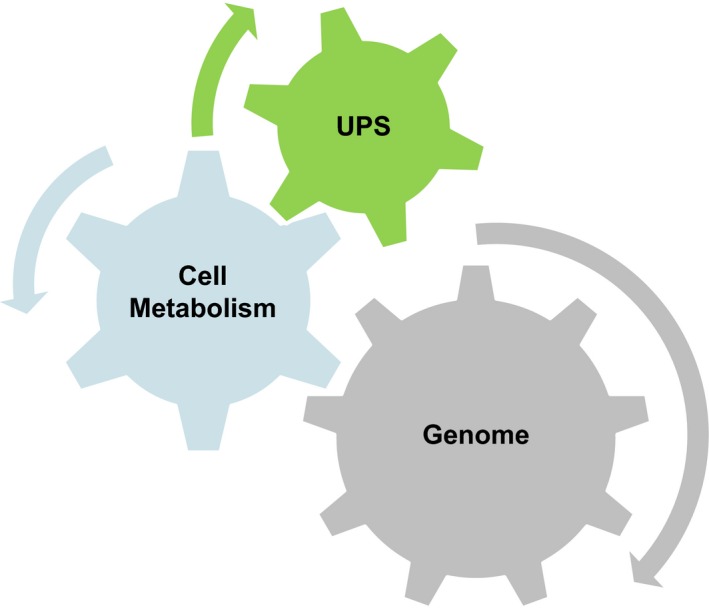
Nuclear proteolysis by the ubiquitin–proteasome system (UPS) regulates the turnover of transcription factors and plays a key role in controlling cell metabolism.

This review focuses on the mechanisms by which the turnover of transcription factors regulates gene activity and metabolism, and how these functions are interconnected (Fig. 1). After a general overview of the UPS, we describe how UPS‐mediated transcription factor degradation dynamically controls chromatin architecture and gene expression. We then highlight the role of the UPS in controlling cell metabolism through mitochondrial gene regulation. We describe the relationship between the corepressor NCoR1 and its E3 ubiquitin ligase Siah2 and the potential of manipulating their mutual regulation to treat metabolic diseases. Finally, we discuss how targeting the UPS may open novel therapeutic options in cancer treatment, using multiple myeloma as an example.

## The ubiquitin–proteasome system

The proteasome and the upstream ubiquitination pathway are an evolutionary conserved and highly regulated proteolytic system that precisely controls selective protein degradation in order to tightly maintain proper protein content and quality. The UPS acts as a disposal pathway and optimizes cellular functions by removing misshaped proteins. The UPS also has a ‘surgical’ role in controlling cell function by adjusting the abundance of regulatory proteins involved in numerous biological pathways, such as signal transduction, stress response, immunity, cell cycle regulation, and transcriptional regulation.

Protein degradation through the UPS pathway is a complex process, involving multiple steps and enzyme machineries that begins when proteins are targeted for degradation by sequential conjugation, usually at lysine residues, to isopeptide‐linked ubiquitin molecules. Ubiquitination is achieved through the coordinated actions of E1 ubiquitin‐activating enzymes, E2 ubiquitin‐conjugating enzymes, and E3 ubiquitin ligases. The interaction between the E3 ubiquitin ligase and its target protein provides a high level of specificity in this dynamic process 3. Hundreds of E3 ubiquitin ligases are encoded by the human genome and the UPS can target a vast number of different proteins for degradation owing to the combinatorial diversity in which ubiquitin ligases and substrate recognition domains cooperate 4. In addition, ubiquitination is a reversible protein modification that can be undone by deubiquitinases, which either trim or fully remove the ubiquitin chains that are attached to substrates, providing an additional layer of regulation to this dynamic process 5.

Once at least four ubiquitin molecules have been conjugated to a substrate in a particular chain formation, the proteasome recognizes the poly‐ubiquitinated proteins as substrates and degrades them 6. The best‐studied types of active proteasomes are formed by the association of a proteolytically active core particle (20S) and 19S regulatory particles, or caps, which create the holo‐proteasome of 26S or 30S with a single or double cap, respectively 7. The regulatory particle controls the activity of the holocomplex by recognizing, unfolding and translocating poly‐ubiquitinated proteins to the 20S protease complex for degradation 8. Intracellular distribution of these proteolytic complexes is dynamic: they can be localized in both the cytoplasm and the nucleus, and unidirectional proteasome transport from the cytoplasm into the nucleus through nuclear pores has been observed 9. Finally, the processed protein substrate is cleaved into short peptide fragments generally 7–9 residues long. Not only is the UPS an elaborate system essential to controlling cellular protein abundance but it also functions as a vital amino acid recycling machine, as it allows reuse of short polypeptides into new protein building blocks 10. In addition, these remnant peptides are loaded onto MHC‐I complexes for presentation on the cell surface, allowing the immune system to survey the proteome composition inside cells 11.

## Dynamic control of gene expression by UPS‐mediated transcription factor turnover

### Transcription factors are generally short‐lived proteins that undergo active turnover

The expression of a gene is not only controlled by the type of transcription factor that binds to its promoter but also by the duration of this interaction. Many transcription factors, particularly those involved in the regulation of cell proliferation, are unstable regulators. Upon completion of their tasks, for instance the recruitment of additional enzymes or cofactors, they are quickly catabolized by the UPS in order to vacate the promoters and allow new regulatory proteins to bind. The stability of the c‐Myc protein, for example, is actively regulated *in vivo* with a half‐life of 20–30 min, and c‐Myc proteolysis is mediated very rapidly by the ubiquitin–proteasome pathway 12.

Interestingly, a global study of the budding yeast proteome categorized half‐lives of proteins based on their function. Based on the integration of proteomic data with previous measurements of mRNA levels and translation rate, two functional clusters of coregulated genes can be distinguished: a first group (‘production cluster’) encodes for abundant and generally stable proteins that appear to be optimized for maximum protein production and maintenance efficiency (ribosomal proteins, proteins involved in protein biosynthesis or enzymes involved in amino acid metabolism). The second group, called ‘regulatory cluster’, is enriched in proteins produced in smaller quantities and with short half‐lives, whose elimination is regulated by the UPS. This second cluster seems to be optimized for regulatory flexibility and therefore mainly consists of cell cycle proteins and transcriptional regulators 13.

The first quantitative genome‐scale prediction of protein abundance and turnover in mammalian cells confirmed a link between protein stability and function 14. Consistent with the genome‐wide studies in *Saccharomyces cerevisiae*, diverse subsets of genes were identified in connection with their enrichment in particular biological and functional processes. Constitutive cellular processes like translation (ribosomal proteins), and central metabolism (glycolysis) were mainly regulated by a first subset of genes with stable proteins, whereas a second group of genes including many transcriptional regulators, signal transduction genes, chromatin modifying enzymes, as well as genes involved in mitosis or cell cycle, encodes short‐lived proteins 14.

### The turnover of transcription factors actively regulates gene expression

The UPS influences transcription through proteolytic and nonproteolytic activities, including regulation of transcription factors and RNA polymerase activity, as well as epigenetic histone ubiquitination 15, 16, 17. Several hypotheses have been proposed about why regulators that activate transcription are degraded 18. The short lifespan of transcription factors enables cells to quickly respond to microenvironmental fluctuations or developmental changes and dynamically adapt gene transcription. By fine‐tuning the local abundance of transcription factors, the UPS can regulate gene expression. Generally, proteasome‐mediated proteolysis of transcription factors allows up‐ or down‐regulation of expression, depending on whether suppressors or activators of transcription are targeted 19, 20.

#### Proteolytic control of transcription factors by the UPS

The concept that the spatio‐temporal regulation of gene expression is influenced by the binding as well as the removal of transcriptional regulators is well established. Ubiquitination of transcription factors and their proteasome‐mediated turnover are crucial steps in this process. A review from the Tansey laboratory suggests two distinct strategies in which cells control the nuclear abundance of transcription factors by ubiquitin‐mediated proteolysis. The first mechanism takes place off chromatin and represents a constitutive turnover in which the transcription factor is maintained in a constitutively unstable form and in restricted abundance by the UPS 3, 20. In this model, the UPS limits the availability and activity of transcriptional activators. When an extranuclear signal arises and a rapid transcriptional response is required, the cell shuts off proteolysis to transiently stabilize and increase transcription factors levels. The regulation of hypoxia‐inducible factor‐1 α (HIF‐1α) by the von Hippel–Lindau tumor suppressor protein (pVHL) is perhaps the best‐known example of this type of transcription control. HIF‐1α is a highly conserved transcriptional regulator indispensable for the cellular responses to reduced oxygen levels 21. Under normoxic conditions, the alpha subunits of HIF are hydroxylated by oxygen‐dependent proline hydroxylases and subsequent pVHL‐mediated ubiquitin proteolysis rapidly degrades HIF‐1α. However, under hypoxia or in pVHL‐defective cells, HIF‐1α hydroxylation and protein degradation are reduced, leading to HIF‐1α stabilization, which associates with HIF‐1β to induce a stress response to hypoxia 22.

The second proteolytic method occurs on chromatin at promoter and enhancer sites and during the process of transcriptional activation 3. It was proposed that kinases associated with the general transcription machinery phosphorylate and inactivate transcriptional activators. This feedback mechanism ensures that activators are shut off after recruitment of the transcriptional complex, in order to limit uncontrolled re‐recruitment of polymerase. Consequently, phosphorylation labels the activator as ‘spent’, preventing it from further activating transcription. Simultaneously, activator phosphorylation also triggers recruitment of the UPS that targets the transcriptional activator for degradation *in situ*, and allows a ‘fresh’ pool of transcription factors to bind to the cleared promoter region and catalyze a new round of transcription 3. A well‐established example of this model of transcription‐coupled destruction, in which ‘kamikaze’ activators are eliminated during transcriptional activation, is the ubiquitin‐dependent elimination of the transcription factors Smad2/3 23. After phosphorylation by the transforming growth factor‐β (TGF‐β) receptor complex, the heterodimer Smads2/3 translocates to the nucleus, where it accumulates and modulates transcription of TGFβ‐target genes. Phospho‐Smads2/3 are poly‐ubiquitinated by the E3 ubiquitin ligase Arkadia after the initiation of gene transcription, coupling degradation of phospho‐Smad 2/3 with the successful activation of target gene transcription, to efficiently terminate signaling at the end of the cascade 24.

#### The UPS is functionally tied to transcription factor activity

Growing evidence supports the concept that the function of a particular transcription factor is more strongly linked to its binding dynamics than to static gene occupancy. It is well established that transcriptional activators are fine‐tuned by the UPS and ultimately turned off after their activation 18. The paradoxical notion that activation and destruction of a transcription factor are linked makes sense in light of a self‐limiting regulatory loop. At the same time, the expression of downstream genes may increase upon transcription factor degradation, as it clears the promoter and allows for rapid binding of new copies of the regulator to facilitate another round of transcription. Examples of transcription factors in which the activation domain and the degradation domain physically overlap include unstable transcription factors such as HIF‐1α, Estrogen receptor α, c‐Myc, or p53 3, 25. Thus, transacting regulatory molecules may be degraded because of their capacity to enhance transcription 25. In their studies on the yeast transcriptional repressor MATα2, Hickey *et al*. show that the ability of a transcription factor to function can be associated with its ubiquitin‐dependent degradation. With a half‐life of only 5 min, MATα2 is a short‐lived protein known to be rapidly ubiquitinated by at least two distinct E3 ubiquitin ligases 26. Its ubiquitin‐mediated proteolysis is required for the expression of genes that control the switch between different mating types in *Saccharomyces cerevisiae*. The authors reported that several mutations within the second degradation target site and homeodomain of MATα2 result in stabilization of this protein, but impair the ability of MATα2 to interact with DNA 27. Another example has been elucidated by the Tansey group on the role of UPS‐mediated proteolysis of Gal4. Elimination of this transcriptional activator in *Saccharomyces cerevisiae* requires the F‐box protein Dsg1/Mdm30 that targets Gal4 for UPS‐mediated proteolysis in cells grown under activating conditions (with galactose). Dsg1/Mdm30‐mediated Gal4 turnover surprisingly stimulates the expression of *GAL* genes, while in Dsg1/Mdm30‐deficient cells, Gal4 stabilization prevents the transcriptional activation of *GAL* genes. The authors concluded that the proteolytic degradation of Gal4 under activating conditions is essential for its activator function 28. In accordance with these findings, the proteasome inhibitor MG132 was shown to inhibit transcription of the Gal4 target genes upon galactose induction 29. These studies, together with work from the Deshaies group, suggest that for some transcription factors, the UPS increases the speed of the cycle of transcription factor binding to DNA, polymerase recruitment, and transcription factor elimination 29. Rapid elimination of ‘spent’ transcription factors resets the promoter and allows new copies of transcription factors to bind. This mechanism ensures that promoters remain receptive to regulatory input. Interestingly, there is evidence that the turnover rate of a given transcription factor varies, depending on the chromatin context in which it resides 30, 31.

### Enrichment of DNA‐associated proteolysis at active genomic regions and involvement of proteolytic degradation in the regulation of chromatin architecture

#### Physical interactions between the UPS and chromatin

Ubiquitin‐mediated proteolysis of activators is a critical component of transcription regulation, as evidenced by the deep physical connections between the transcription machinery and the UPS. First, ubiquitin ligases have been demonstrated to interact with genomic sites of activator function. Von der Lehr *et al*. 32 observed an association of the F‐box protein Skp2, ubiquitinated proteins, and subunits of the proteasome with a c‐Myc target promoter *in vivo*. Another example of how the UPS closely impacts transcription is the direct protein–protein interaction of the yeast Asr1 ubiquitin ligase with the carboxy‐terminal domain of RNA polymerase II. Ubiquitination by Asr1 alters the subunit composition of the enzymatic complex and is associated with inactivation of polymerase function 33. Similarly, the E3 ubiquitin ligase Rsp5 targets the largest subunit of RNA polymerase II *in vitro* 34. Also, there is evidence for selective recruitment of either E3 ubiquitin ligases CHIP or MDM2 to the PSA promoter depending on the androgen receptor (AR) phosphorylation status, which therefore directs ubiquitin‐dependent degradation of AR at the PSA promoter. AR phosphorylation is a key regulatory step for the recruitment of MDM2 which targets AR for proteosomal degradation. When AR phosphorylation is impaired, CHIP is recruited instead of MDM2, and ensures the continuity of AR degradation through the recruitment of the UPS machinery at the promoter of activated genes to degrade AR 35.

Furthermore, the proteasome is intimately involved in the regulation of gene expression. This notion is supported by chromatin immunoprecipitation (ChIP) studies in yeast showing that proteasome subunits actually bind to chromatin 36. For instance, the 19S proteasome regulatory particles are mobilized to the active *GAL1* promoter by the Gal4 transactivator upon induction with galactose 16. Likewise, a subset of 19S proteasome proteins is recruited to the promoters of ribosomal protein genes RPS5, RPL2B, and RPS11B *in vivo*, and promotes the association of a specific coactivator/histone acetyl transferase in order to facilitate the recruitment of TFIID for transcriptional initiation 37. More recently, ChIP assays using highly specific antibodies for native yeast proteasome subunits demonstrated that not only components of the 19S regulatory particle but also the major 20S proteolytic core associate with the activated *GAL10* gene in a similar temporal and spatial manner 38. Moreover, in a genome‐wide study in *Saccharomyces cerevisiae*, ChIP combined with global transcriptome analyses identified genomic regions regulated by the proteasome, providing evidence that proteasome association correlates with a vast array of highly transcribed genes 36. The association of the entirety of components of the UPS (i.e., ubiquitin ligases and 26S canonical proteasome complexes) with active genomic regions highlights the physical and functional connections between transcriptional regulation and protein turnover.

Even though proteolysis and transcription are functionally interconnected and might be key determinants of cellular metabolism, the genome‐wide degradation pattern of transcription factors is still poorly understood. Chromatin immunoprecipitation assays are widely used to produce a static snapshot of transcription factor occupancy at specific DNA regulatory sequences, but they fail to reflect the transcription factor‐binding dynamics across the genome 39. Despite the well‐established link between transcription and proteasome‐mediated proteolysis, most of the studies on how the UPS regulates transcription focus on substrate specificity, but do not define the chromosomal location of substrates targeted by the proteasome. In accordance with earlier findings in yeast, genome‐wide studies of DNA‐associated proteolysis in mouse and human cells by our group confirmed a tight connection between degradative ubiquitination and activity of promoters and enhancers 31. Using a combination of ubiquitin chromatin immunoprecipitation followed by next‐generation sequencing (ChIP‐seq) and functional analyses, we demonstrated that DNA‐associated degradation is enriched at genes with high transcriptional activity in mammalian cells. These results suggest that localized transcription factor turnover represents a key regulatory step in the continuous adjustment of gene expression in lower as well as in higher eukaryotes.

#### Regulation of chromatin structure by the UPS

Proteolytic activities of the proteasome occur in the immediate vicinity of DNA and participate in accessing, modifying, or controlling chromatin in order to regulate transcription. Different models for recruitment of proteasome subunits to chromatin have been proposed. Active proteasomes can be connected to the chromatin by direct contact with activators; indirectly through their interactions with intermediate proteins, or they can also bind to chromatin in response to the presence of ubiquitinated substrates at specific loci 40. Recent work from the Rosenfeld laboratory revealed a role of proteasome‐dependent degradation in enhancer looping 41. This study showed that condensins, proteins involved in chromatin architecture, are abundant at ERα‐bound active enhancers. Upon ligand‐induced activation of ERα, condensins recruit the E3 ubiquitin ligase HECTD1 to remove repressors, increase expression of enhancer RNA and promote enhancer/promoter looping to fully activate transcription in target genes. The proteasome is also mobilized to DNA through its direct modulation of the chromatin composition. This has been shown by the dual roles of the conserved Uch37 deubiquitinating enzyme. As a component of the 19S regulatory particle, it strips off poly‐ubiquitin chains from proteins so that they can enter into the narrow proteolytic 20S core for degradation. In addition, Uch37 is associated with the Ino80‐like chromatin‐remodeling complex (Ino80) 42. After binding to promoter regions of certain actively transcribed genes, the Ino80 nucleosome remodeler reorganizes chromatin structure by catalyzing nucleosome sliding during transcription 43. While a member of the Ino80 chromatin‐remodeling complex, Uch37 is strongly inhibited and kept in an inactive state, but Uch37 can be reactivated as a deubiquitinase through dynamic interaction of the Ino80 complex with the proteasome 42. Taken together, these observations illustrate that the UPS and components of the chromatin‐remodeling complexes can cooperate to regulate transcription.

## Regulation of cellular energy metabolism through protein degradation

### Activation through de‐repression: the key role of the corepressor NCoR1 in mitochondrial gene regulation

Like transcriptional coactivators, transcriptional repressors and corepressors are also restricted by the UPS and failure to eliminate repressors results in aberrant transcriptional silencing. When mapping DNA‐associated protein turnover on a genome‐wide scale, we found that promoter‐linked proteolysis has a positive effect on the transcription of a significant number of genes, one of the most prevalent groups being the nuclear‐encoded mitochondrial genes 31. Promoter regions of many of these genes contain DNA‐binding motifs for the transcriptional activator cAMP response element‐binding protein (CREB), which plays an important role in modulating gene transcription in response to metabolic changes. We identified the nuclear receptor corepressor 1 (NCoR1 or TRAC1) as a protein partner of CREB at the promoters of proteasome‐sensitive genes 31. Interestingly, NCoR1 seems to be a target of choice for the UPS, specifically when bound to promoters of nuclear‐encoded mitochondrial genes 31, 44, 45. NCoR1 is well‐characterized for its role as transcriptional corepressor of nuclear hormone receptor target genes. In the absence of ligand, the N‐terminal‐interacting region of NCoR1 recruits histone deacetylases (predominantly HDAC3) while the C‐terminal domain binds to the unliganded (i.e., not activated) transcription factor (nuclear hormone receptor) to repress transcriptional activity 46, 47, 48. Upon binding of ligand, conformational changes in the transcription factor dislodge NCoR1 and license it for degradation. The corepressor is then replaced by a coactivator, such as peroxisome proliferator‐activated receptor γ coactivator 1 α (PGC‐1α), and gene expression ensues 44. At this point, we can only speculate that a similar cofactor exchange occurs at metabolic genes that are under control by NCoR1 and CREB, but it is noteworthy that several mitochondrial subunits of the electron transport chain complex are up‐regulated in response to PGC‐1α induced transcription 49. In skeletal muscle, NCoR1 antagonizes PGC‐1α and represses genes involved in oxidative phosphorylation 50, 51. In accordance with these findings, muscle‐specific NCoR1^−/−^ mice exhibit enhanced muscle mass and oxidative capacity of muscle fibers, which present a higher mitochondrial content and activity 51. In conclusion, the UPS‐mediated proteolytic switch that activates mitochondrial gene promoters constitutes a major regulator of biogenesis and coordinates the maintenance of metabolic homeostasis.

### Mitochondrial retrograde signaling is involved in the regulation of cancer metabolism

Mitochondria play a central role in the metabolism of most eukaryotic cells. Mitochondrial dysfunction has been implicated in the development of a variety of pathological conditions and illnesses, including not only age‐related neurodegenerative diseases such as Alzheimer's dementia, Parkinson's disease, or amyotrophic lateral sclerosis, but also type 2 diabetes and cancer 52, 53. Specifically, cancers have been shown to effectively reprogram metabolism by switching mitochondrial function and favor the production of molecular building blocks over ATP generation 54. Historically, Otto Heinrich Warburg first described that tumor cells exhibit increased aerobic glycolysis compared to normal cells, then proposing that dysfunctional mitochondrial respiration may be a cause of this phenomenon (‘Warburg effect’). However, as many tumors preferentially generate energy by metabolizing glucose to lactic acid while retaining effective mitochondrial respiration, new concepts of cancer cell metabolism have emerged. The goal of these studies is to explain how mitochondrial reprogramming affects cell cycle, gene expression, metabolism, and tumor cell survival, and therefore promotes cancer cell growth and tumorigenesis 55.

The majority of genes encoding mitochondrial proteins is located in the nucleus 56. There is clear evidence that mitochondria communicate to the cell nucleus through a retrograde response 53. Retrograde signaling from the mitochondria to the nucleus is a complex mechanism that controls the expression of nuclear genes in response to changes in the functional state of mitochondria and the metabolic state of the cell. The retrograde response may be well established in yeast, but remains ill‐defined in more complex eukaryotes 57. Cancer‐related mitochondrial events have direct regulatory consequences on nuclear gene expression through their impact on a variety of key signaling pathways. For example, oncogene‐induced accumulation of reactive oxygen species (ROS) in cancer cells, produced primarily by defective mitochondria, promotes cancer cell growth and survival. Excessive formation of mitochondrial ROS stabilizes HIF‐1α and activates the nuclear respiratory factor‐2 (NRF‐2), FOXO, and NF‐κB transcription factors, and deactivates tumor‐suppressive enzymes such as PTEN, MAP‐kinases, and caspases 58.

The UPS is tightly linked to mitochondrial function. Degradation events control the fusion and fission cycle of these organelles, trigger mitophagy, and also eliminate misfolded or mislocalized mitochondrial proteins 59, 60, 61, 62. Given these interconnections, it is not surprising that mitochondria also have the capacity to regulate the UPS. It was recently shown that oxidative and metabolic stress affects ubiquitin‐dependent proteolysis. A study in *C. elegans* demonstrated that dysregulation of mitochondrial respiratory complexes induces elevated levels of mitochondria‐derived ROS 63. The resultant oxidative stress correlated with impaired UPS activity and resulted in proteolytic failure in the cytosol. Similarly, ubiquitin‐dependent protein turnover defects were also observed in primary cell lines derived from patients affected by the mitochondrial disease isovaleric acidemia. The turnover defects were linked to higher oxidative stress in these patients’ cells caused by defective mitochondrial metabolism 63. These observations establish that mitochondrial metabolism can modulate the efficiency of UPS pathways in the cytosol, and suggest that ROS‐induced down‐regulation of UPS activity might contribute to the progression of human mitochondrial diseases by attenuating the cellular protein quality control systems or by altering UPS‐mediated transcriptional regulation.

Among other key signaling events triggered by dysfunctional mitochondria is the altered production of metabolites that have an effect on the epigenetic code. Components such as α‐ketoglutarate, fumarate, or succinate can modify the methylation status of histones and DNA, and alter the stability of transcription factors through prolyl‐hydroxylases 53, 64, 65, 66. For instance, demethylases are α‐ketoglutarate‐dependent and a reduction in the production of this essential cofactor by the Krebs cycle or production of ‘competitive oncometabolites’ in cancer cells, leads to enzymatic inhibition of the TET2 DNA demethylase and JmjC histone lysine demethylase, resulting in hypermethylation and oncogenic transcriptional changes 65, 66, 67. By extension, communication with the nucleus through metabolic intermediates allows mitochondria to reorganize the chromatin structure and adjust gene expression patterns to accommodate increasing metabolic demand associated with the high proliferation of malignant cells.

### The E3 ubiquitin ligase Siah2 derepresses NCoR1

The E3 ubiquitin ligase seven in absentia 2 (Siah2), together with proteins of the NCoR1 corepressor complex, such as transducin β‐like 1 (TBL1) and TBL‐related 1 (TBLR1) 45, 68, appears to be one of the main factors responsible for ubiquitination of NCoR1. Siah2 has been primarily implicated in the maintenance of normal homeostasis and in response to multiple forms of cellular stress, including hypoxia, variations in glucose levels, DNA damage, and apoptosis. Siah2 has also been extensively studied in pathologies associated with fluctuating oxygen tension and HIF‐1α activity 69. Siah2 increases stability of HIF‐1α through degradation of negative HIF‐1α regulators. This ubiquitin ligase thus has opposing effects on mitochondrial function, by either derepressing nuclear‐encoded mitochondrial genes through NCoR1 degradation, or by repressing them through stabilization of the hypoxia factor HIF‐1α. How these functions are differentially regulated is still unclear. Siah2 protein expression is markedly up‐regulated in human high‐grade breast cancer, lung cancer, and castration‐resistant prostate cancer, suggesting a contribution to tumorigenesis 70, 71. A role of Siah2 in tumor formation and metastasis has already been established in genetically engineered mouse models. For instance, homozygous Siah2 knock‐out mice crossed with the TRAMP mouse model of prostate cancer showed that the formation of neuroendocrine prostate tumors is Siah2‐dependent 72. In addition, depletion of Siah2 by siRNA in human hepatocellular carcinoma decreases cell proliferation and motility, as well as sensitizes the cells to cytostatic treatment 73. Another function of Siah2 is the degradation of tumor‐suppressors, thus contributing to cell transformation. Upon tyrosine phosphorylation by Src, Siah2 interacts with CCAAT/enhancer‐binding protein delta (C/EBPδ), a tumor suppressor that is down‐regulated during breast cancer progression, and promotes its poly‐ubiquitination and proteasomal degradation. Src/Siah2‐induced inhibition of C/EBPδ expression leads to increased cyclin D1 expression, enhanced invasive properties, and proliferation of breast epithelial cells 74.

In addition, a recent study identified a role of Siah2 in the development and progression of castration‐resistant prostate cancer 71. By selectively ubiquitinating a subset of ARs that are bound to the corepressor NCoR1, Siah2 initiates the removal of this pool of transcriptionally inactive ARs from their *cis*‐sequences and allows replacement by p300‐bound active ARs. This example highlights the complexity of nuclear degradation, which is not only protein‐specific but also specific in terms of the chromatin location at which it occurs.

**Figure 2:**
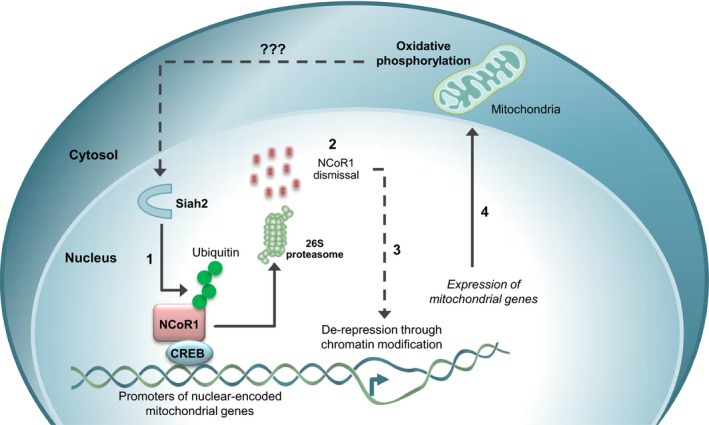
Proteasome‐dependent de‐repression of metabolic genes. Nuclear receptor corepressor 1 (NCoR1) is a target for degradation by the ubiquitin–proteasome system (UPS), especially at promoters of nuclear‐encoded mitochondrial genes. The E3‐ubiquitin ligase Siah2 is responsible for NCoR1 ubiquitination (1). NCoR1 dismissal (2) from the transcription factor cAMP response element‐binding protein (CREB) increases transcriptional activity at metabolic genes, possibly by allowing coactivators to bind to CREB (3). H3K27 acetylation levels increase upon NCoR1 elimination and stimulate the expression of mitochondrial genes, especially those encoding components of the respiratory chain and of substrate transporters (4) 31. We have observed rapid up‐regulation of Siah2 and removal of NCoR1 from nuclear mitochondrial promoters upon inhibition of mitochondrial activity (not shown). Licensing of NCoR1 elimination from these genomic sites may be a key element of retrograde signaling between mitochondria and the nucleus. How exactly UPS activity is regulated in this particular transcriptional context remains to be elucidated.

Given the importance of Siah2 in the regulation of mitochondrial metabolism and its tumor‐promoting role, it is worth mentioning that the NCoR1/HDAC3 repressor complex also controls cell proliferation *via* its role in cell cycle regulation and DNA repair pathways. Bhaskara *et al*. 75 found that a vast proportion of human hepatocellular carcinoma has reduced NCoR1 expression levels. More specifically, the NCoR1/HDAC3/SMRT complex participates in the maintenance of acetylation and methylation of cell cycle‐associated histone marks during S‐phase progression of cell cycle, which is crucial for the DNA damage response, maintenance of chromatin structure, and genome stability 75. Siah2‐mediated ubiquitination targets NCoR1 for rapid destruction by the proteasome 45. Proteolysis of NCoR1 derepresses target genes, as evidenced by the increase in histone H3K27 acetylation upon NCoR1 dismissal 31. Especially with the prospect of pharmacologically controlling metabolism, mechanisms such as the continuous elimination of NCoR1 offer the potential to alter mitochondrial respiratory activity (Fig. 2). In conclusion, these observations suggest that the Siah2/NCoR1 axis may be an attractive target for the treatment of metabolic diseases and several types of cancers.

## Targeting the UPS in hematological malignancies

### Inhibition of the proteasome as treatment in multiple myeloma

Given its fundamental and ubiquitous role in maintaining cellular homeostasis, it is perhaps surprising that pharmalogical inhibition of the proteasome can be effective in the treatment of cancer, with relatively limited general side effects. Historically, proteasome‐targeted therapy has first shown positive results in the treatment of the hematological malignancy multiple myeloma (MM) 76, 77. In this disease, B cell‐derived transformed plasma cells clonally multiply and accumulate in the bone marrow, where they generate and secrete high amounts of monoclonal antibodies. The results are lesions of the bone, dysfunctional blood flow and immunity due to circulating antibodies, and displacement of healthy hematopoiesis. Bortezomib, a small molecule inhibitor of the 26S proteasome was approved by the FDA in 2003 as the first proteasome inhibitor for the treatment of relapsed or refractory MM in patients and marketed under the brand name Velcade. At a molecular level, this boronic dipeptide predominantly and reversibly inhibits the chymotrypsin‐like activity of the β5‐subunit of the proteasome, although it also interferes to a lesser extent with the activity of the β1 subunit 78. As Bortezomib shows selective cytotoxicity to cancer cells compared to normal cells, clinical introduction of Bortezomib, either alone or in combination with other therapies, has resulted in extended survival times of patients with MM, mantle cell and follicular lymphoma 79. However, the median survival of patients remains very low at ~ 4–6 years. Indeed, although the vast majority of MM patients initially respond to Bortezomib therapy, a residual subset of cells that are resistant to the original therapy usually causes the tumors to relapse quickly and many patients become insensitive to treatment. Therefore, overcoming drug resistance and developing innovative proteasome inhibitors with less peripheral side effects such as neuropathies remain some of the future challenges for MM treatment.

Carfilzomib, the second FDA‐approved proteasome inhibitor for treating relapsed and resistant MM previously treated with Bortezomib, has improved proteasome‐targeting selectivity and efficacy, with a lower toxicity profile 80. Phase I–III clinical trials involving patients with previously treated MM and non‐Hodgkin's lymphomas have demonstrated more potent clinical and fewer off‐target effects. Additional second‐generation proteasome inhibitors, such as Marizomib, Ixazomib, Delanzomib, Oprozomib, and a variety of natural products are also being tested in phase I and phase II clinical trials for the treatment of recurrent multiple myeloma, lymphomas, leukemias, and solid tumors. Some of these compounds display different pharmalogical and chemical properties than Bortezomib. For example, Marizomib is able to block the chymotrypsin‐like activity of the proteasome in an irreversible manner and also inhibits the caspase‐like and trypsin‐like activities of the catalytic β‐subunits.

However, one of the major remaining questions is how proteasome inhibitors actually kill MM cells. Knowing which pathway disruption is critical to MM treatment would help us devise more specific modes of intervention that target a defined E3 ligase rather than use ‘blunt’ proteasome inhibition. Originally, the proposed mode of action of Velcade was to block inflammatory signals that are critical for MM cells by stabilizing the NF‐κB inhibitor IκB. However, Velcade actually seems to stimulate this pathway 81. Gene expression profiling in cell lines shows that Velcade treatment predominantly suppresses nuclear‐encoded mitochondrial genes. In particular genes associated with the mitochondrial respiratory complex and with mitochondrial transporters appear to be susceptible to this treatment [82, and data not shown]. This raises the possibility that stabilization of the corepressor NCoR1 at these genes is at least partially responsible for the impact of proteasome inhibition on MM cells. Indeed, biosynthetic pathways are among the ones that are most strongly suppressed by proteasome inhibition. This type of treatment may hit tumors with two punches: choking off energy supply by repressing mitochondria through NCoR1 and dialing down the translation machinery by triggering the unfolded protein response 83. MM cells are uniquely predisposed to these stresses due to the fact that they are hardwired to produce and secrete antibodies at no benefit, a process that is immensely energy demanding.

**Figure 3:**
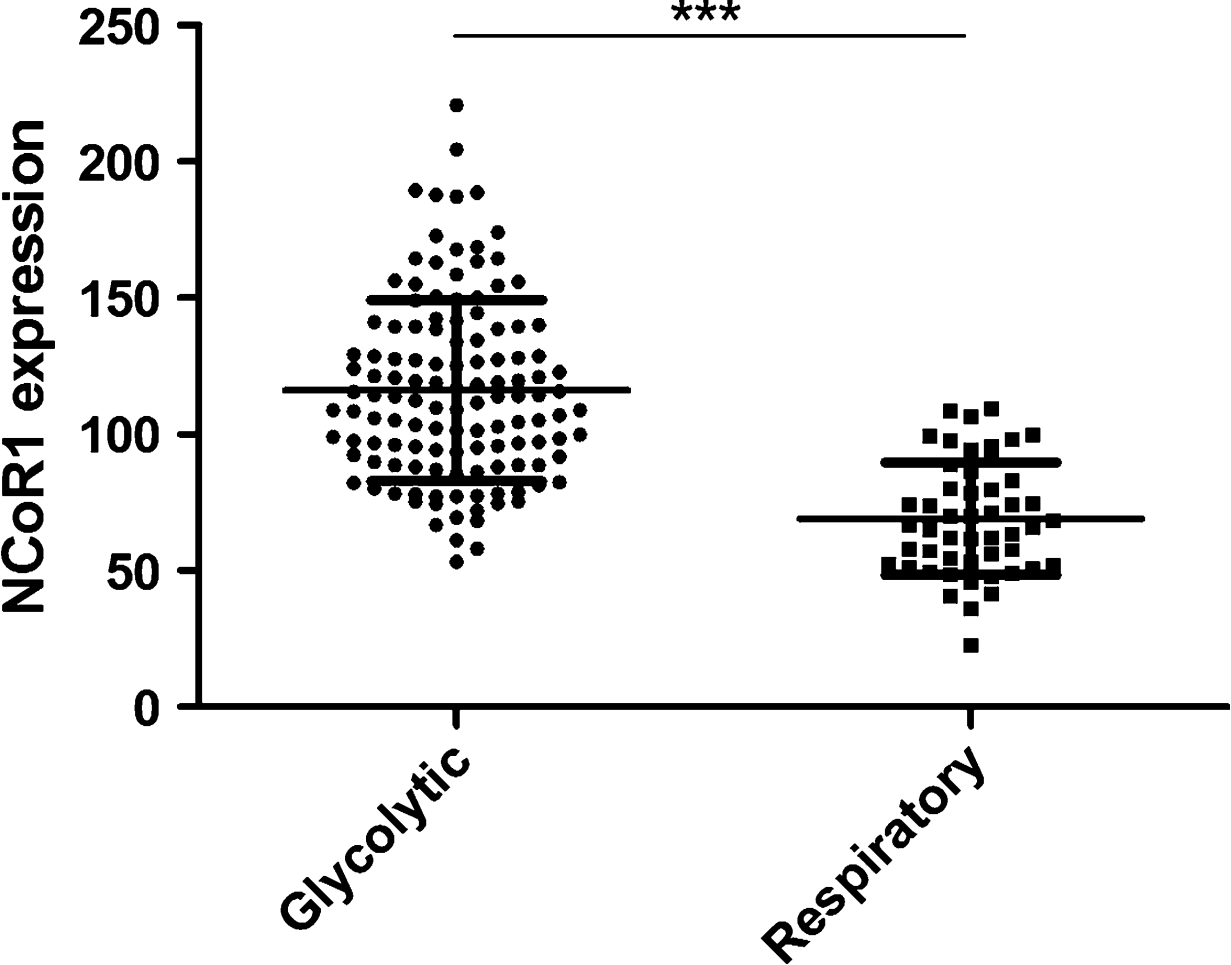
Diffuse large B‐cell lymphoma (DLBCL) can be divided based on metabolic profiling. Nuclear receptor corepressor 1 (NCoR1) mRNA expression correlates with the metabolic signature in DLBCL, suggesting a function as master repressor of oxidative phosphorylation. NCoR1 expression is 1.7‐fold higher in the glycolytic subset of DLBCL compared to the respiratory subset (*P* < 0.0001). *Y*‐axis denotes transcript expression in arbitrary units. Figure based on raw data available from the Broad Institute's Cancer Program Legacy Resource.

**Figure 4:**
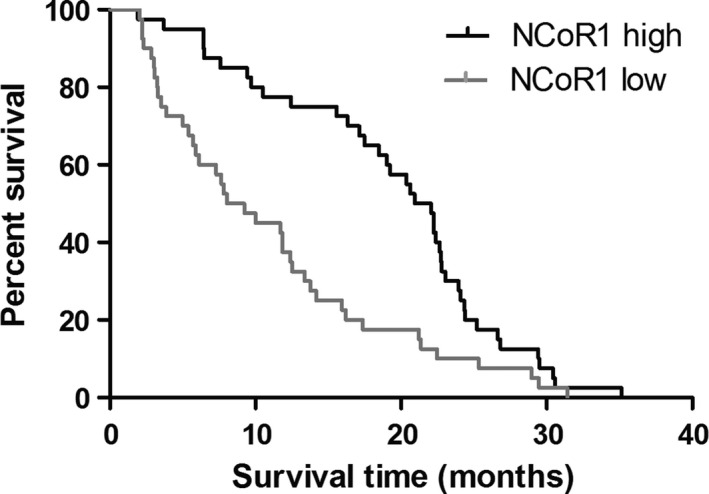
High levels of nuclear receptor corepressor 1 (NCoR1) expression are associated with a better survival of multiple myeloma patients treated with the proteasome inhibitor Velcade. Multiple myeloma (MM) patients with high expression of NCoR1 (and repressed mitochondrial signature) have a survival benefit when treated with Velcade (*P* < 0.0008), but not when treated with Dexamethasone (not shown). Median survival is 2.5‐fold higher in patients with high NCoR1 expression when compared to patients with low NCoR1 expression. The analysis is based on gene expression profiling of MM cells in 264 patients prior to treatment 85.

Diffuse large B‐cell lymphoma (DLBCL) is a malignancy that can be differentiated based on its metabolic profile into a ‘respiratory’ and a ‘nonrespiratory’ subgroup 84. In these groups, we found expression levels of the mitochondrial repressor NCoR1 to be low and high, respectively (Fig. 3), suggesting that this factor indeed dictates mitochondrial activity. Interestingly, levels of positive mitochondrial regulators such as NRF‐2 and PGC‐1α do not differ significantly between the two groups (data not shown). NCoR1 expression is also inversely correlated with the expression of mitochondrial genes in MM patients (data not shown). Given the susceptibility of the NCoR1‐controlled transcriptome to proteasome inhibition, we further investigated the connection between Velcade sensitivity and NCoR1 expression. Remarkably, high expression of NCoR1 (and repressed mitochondrial signature) is associated with a favorable response to Velcade treatment (Fig. 4). This survival benefit is not evident in patients who were treated with Dexamethasone (data not shown). We do not yet fully understand this observation, but one possibility is that tumor cells with high NCoR1 levels and repressed mitochondrial activity already grow at their metabolic limit. Hitting these cells with a drug that further decreases oxidative metabolism may limit cell growth and contribute to the survival benefit observed in patients with high NCoR1 levels in their cancer cells.

Gene expression profiling and correlation with outcome in clinical trials for Velcade in MM patients shows that many of the pathways associated with progressive disease and response to treatment regulate protein biosynthesis and mitochondrial functions. Results of Velcade chemotherapy in solid tumors, including lung, breast, and prostate cancers, have been somewhat disappointing. However, as the uncontrolled synthesis of antibodies and the associated high energy demand increases their dependence on mitochondrial activity, MM cells may be more sensitive to metabolic treatments 85, 86, 87. NCoR1 has also been linked to several types of leukemia. For instance, a recent integrative genomic analysis of chronic myelogenous leukemia patients treated with Imatinib (a tyrosine kinase inhibitor) showed that NCoR1‐targeted gene signatures were significantly associated with sensitivity to Imatinib. Moreover, the study revealed that Imatinib particularly targeted the NCoR1 governed transcriptome, suggesting that other cancers might also benefit from treatments that stabilize NCoR1 88.

### Therapeutic perspectives for E3 ubiquitin ligases as targets in cancer treatment

Beyond its impact on basic molecular and biological processes, site‐selective transcription factor degradation also holds great medical promise. Transcription factors are generally deemed ‘undruggable’. The ability to modify transcription factor‐specific E3 ubiquitin ligases with small molecules would allow us to facilitate or block the elimination of DNA‐binding regulatory proteins. Because the process of ubiquitination is located upstream of the proteasome and because E3 ubiquitin ligases mediate the specificity of substrate ubiquitination, small molecule modifiers of these enzymes represent more specific and valuable drug candidates than therapies based on proteasome inhibition. Small molecules that inhibit or stimulate specific E3 ubiquitin ligases represent the most appealing target for drug development and are currently being tested for utility in hematological malignancies 80. In fact, one such ubiquitin ligase modulator is already part of the front‐line treatment in MM. The drug Thalidomide targets the E3 ubiquitin ligase Cereblon. This causes the E3 ubiquitin ligase to alter its substrate specificity and ubiquitinate two B cell‐specific transcription factors, Ikaros family zinc finger proteins 1 and 3 (IKZF1 and IKZF3), which are critical for the survival of MM cells. Once ubiquitinated, the transcription factors are quickly removed by the proteasome and the loss of IKZF1 and IKZF3 is in part responsible for the cytotoxic effect of Thalidomide in MM cells. This example shows how the UPS can be directed by small molecules to selectively remove specific target proteins.

Another example of a promising therapeutic strategy is the targeting of the E3 enzyme Mdm2. Inhibitors of the ubiquitin ligase are being tested, as the enzyme plays an essential role in regulating p53 turnover. Not only does Mdm2 promote proteasomal degradation of p53, but it also inhibits the transactivation domain of the tumor suppressor protein 89. The tumor suppressor protein p53 is inactivated in approximately 50% of human cancers. Aside from p53 mutations, the main inhibitory mechanism of p53 has been shown to be the overexpression of its negative regulator Mdm2 through gene amplification, increased transcription or translation. Overexpression of Mdm2, and the subsequent elimination of p53, is a main determinant in promoting both the proliferation and tumor cell survival of multiple myeloma cells by reducing p53 protein levels 89, 90. Inhibition of Mdm2 results in p53 reactivation and represents an attractive potential treatment strategy for this disease. Considering the fact that newly diagnosed cases of MM rarely carry inactivating p53 mutations, inhibitors of p53‐Mdm2 interactions are currently being developed in clinical trials. Nutlins, a group of cis‐imidazole analogs, antagonize the interaction between Mdm2 and p53 by binding with high affinity to the p53‐binding pocket of Mdm2 and competitively displacing p53 80. Stabilization and accumulation of p53 protein subsequently causes reactivation of its downstream pathway in cancer cells with functional p53. For instance, nutlin‐3 can induce apoptosis in cell lines harboring either wild‐type or mutant p53, deriving from hematological malignancies such as MM, ALL, AML, CLL, and Hodgkin's lymphoma 91.

Recently, new roles have emerged for FBXW7, a substrate‐targeting subunit of the SCF (Skp1‐Cul1‐Fbox) ubiquitin ligase complex, in leukemia. Indeed, a comprehensive screen of FBXW7 mutations in various human malignancies detected that more than 30% of T‐cell acute lymphoblastic leukemia (T‐ALL) patients carry FBXW7 mutations. As FBXW7 mediates the ubiquitin‐dependent proteolysis of several oncoproteins including c‐Myc, cyclin E1, c‐Jun, and Notch, FBXW7 is considered a tumor suppressor. In particular, FBXW7 plays a pivotal role in maintaining quiescence in the small pool of leukemia‐initiating cells by mediating c‐Myc degradation 92. FBXW7 deficiency stabilizes c‐Myc and abrogates quiescence and activates leukemia‐initiating cells, a subpopulation of cells that is essential for the propagation of leukemia 93. Moreover, FBXW7 regulates glucocorticoid response in T‐ALL by ubiquitinating the glucocorticoid receptor (GR) and targeting it for proteasome‐dependent degradation. FBXW7 deficiency induces GR stabilization, and enhances the expression of GR target genes, in particular proapoptotic genes such as Bim or PUMA 94.

If NCoR1 is indeed a critical substrate of the proteasome and a determinant of the sensitivity to proteasome inhibitors, it would be desirable to have drugs that specifically block its degradation. Given the importance of the E3 ubiquitin ligase Siah2 in the regulation of NCoR1, it is reasonable to consider Siah2 as a target of interest in MM 95, 96.

In conclusion, these observations define E3 ubiquitin ligases as the most attractive druggable components of the UPS. It is possible to identify drugs that either stimulate or inhibit specific E3 ligases and thereby degrade or stabilize target transcription factors. Given the selective sensitivity of transcription factors to degradation at certain target genes, one would expect such drugs to interfere with the expression of defined gene sets. These drugs would likely be more effective and feature less side effects than proteasome inhibitors and moreover also have the potential capacity to increase degradation as shown with Thalidomide.

## Conclusion

**Figure 5:**
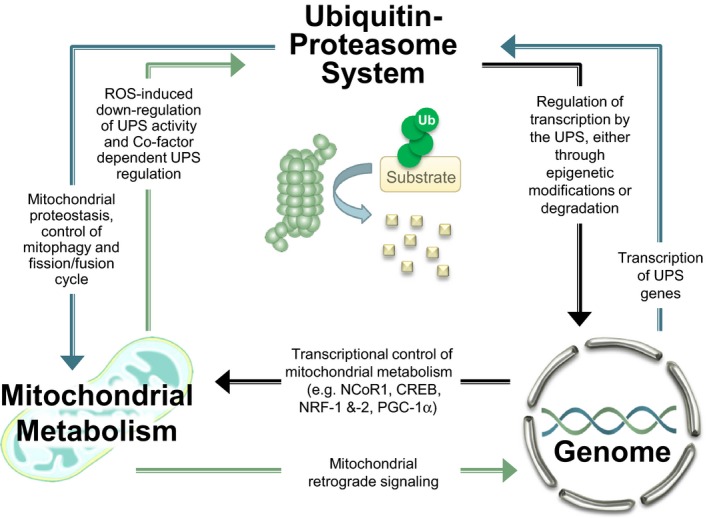
The ubiquitin–proteasome system (UPS) integrates genomic and metabolic function in a dynamic network. Multiple interconnections exist between the UPS, mitochondria, and the genome. Each component influences the other through several regulatory processes that are disturbed in metabolic diseases and cancer. Metabolism controls the availability of energy, but also generates cofactors and metabolites that interfere with UPS activity, such as reactive oxygen species (ROS) and free calcium 57, 97. One of the key remaining questions is how mitochondria communicate with the nucleus to adjust the expression of mitochondrial genes.

Transcription factors are short‐lived proteins under constant surveillance by the UPS. As such, protein turnover is a critical component of genome regulation. Metabolic genes are continuously being adjusted and it is no surprise that these two highly connected systems, degradation and transcription, converge in the control of mitochondrial genes (Fig. 5). The complex interplay between these two pathways also affords us with the exciting prospect of ‘drugging’ transcription by interfering with the turnover of transcription factors at the chromatin level. Manipulating the UPS to degrade or stabilize specific transcription factors at localized genomic sites, and thereby alter distinct genetic programs, might represent an alternative treatment of dysregulated cell metabolism. Given the central role of mitochondria in cancer, metabolic diseases, and aging, it will be interesting to decipher further details of the communication between the mitochondrial and the nuclear compartment and to explore potential new therapies in the treatment of relevant diseases.

## Author contributions

LM and AC co‐wrote the manuscript.
